# Solar radiation, temperature and the reproductive biology of the coral *Lobactis scutaria* in a changing climate

**DOI:** 10.1038/s41598-022-27207-6

**Published:** 2023-01-05

**Authors:** Jessica Bouwmeester, Jonathan Daly, Nikolas Zuchowicz, Claire Lager, E. Michael Henley, Mariko Quinn, Mary Hagedorn

**Affiliations:** 1 Smithsonian Conservation Biology Institute, Front Royal, VA 22630 USA; 2 Hawaiʻi Institute of Marine Biology, Kāneʻohe, HI 96744 USA

**Keywords:** Cell biology, Climate-change ecology, Zoology

## Abstract

Coral reefs worldwide are at risk due to climate change. Coral bleaching is becoming increasingly common and corals that survive bleaching events can suffer from temporary reproductive failure for several years. While water temperature is a key driver in causing coral bleaching, other environmental factors are involved, such as solar radiation. We investigated the individual and combined effects of temperature, photosynthetically active radiation (PAR), and ultraviolet radiation (UVR) on the spawning patterns and reproductive physiology of the Hawaiian mushroom coral *Lobactis scutaria*, using long-term experiments in aquaria. We examined effects on spawning timing, fertilisation success, and gamete physiology. Both warmer temperatures and filtering UVR altered the timing of spawning. Warmer temperatures caused a drop in fertilisation success. Warmer temperatures and higher PAR both negatively affected sperm and egg physiology. These results are concerning for the mushroom coral *L. scutaria* and similar reproductive data are urgently needed to predict future reproductive trends in other species. Nonetheless, thermal stress from global climate change will need to be adequately addressed to ensure the survival of reef-building corals in their natural environment throughout the next century and beyond. Until then, reproduction is likely to be increasingly impaired in a growing number of coral species.

## Introduction

Global climate change has pressed coral reefs around the world into an era of challenge and uncertainty. Coral reefs are among the oldest ecosystems on earth but are also some of the most vulnerable to climate change. They are habitats for a quarter of all marine life, they protect our coastlines and homes, and they feed over one seventh of the Earth’s human population^1,2^. However, they live at the upper limit of their thermal threshold, making them sensitive to even minimal increases in ocean temperatures^3^. Summer temperature anomalies in the last decades have regularly disrupted the fragile symbiosis between corals and their algal symbionts^4,5^, causing coral bleaching, with recurrent and stronger bleaching events expected in the coming decades. Climate change is altering coral reefs around the world, affecting the distribution, abundance and biodiversity of coral reef–associated organisms^6^. In order for corals to survive through the next decades, they must adapt their physiology and metabolism to new ocean circulation patterns, nutrient inputs, and oxygen contents, higher water temperatures, and lower pH^7^. Acclimation to environmental change can occur to some extent via phenotypic plasticity, but lasting, genetic adaptation can come about largely by way of sexual reproduction and natural or selective breeding^8–10^.

Today, most reefs have experienced some degree of bleaching due to climate change^11,12^ and global restoration efforts strive to protect surviving reefs and restore damaged ones^13–17^. During bleaching, the coral hosts are deprived of their nutrient-providing algal symbionts (family Symbiodiniaceae) and slowly starve. If the surrounding environment returns to favourable conditions and the algal symbiont communities repopulate their hosts in time, the corals can recover. However, their overall fitness remains affected, with potentially detrimental long-term impacts. Typically, during coral bleaching, algal symbiont density and chlorophyll concentration are greatly decreased, leading to a drop in symbiont photosynthesis, decreased coral respiration and calcification, and a decline in coral lipids, carbohydrates, protein, and tissue biomass^18–22^. While metabolic processes such as algal symbiont photosynthesis and coral respiration can return to their pre-bleaching levels within 1–3 months once the source of bleaching is removed, in some species, energy reserves such as carbohydrates and proteins may require up to a year to recover^21,23^. Without the necessary energy reserves during the 6 to 11-month gametogenesis cycle observed in most scleractinian coral species^24–26^, coral reproduction can remain disturbed for a prolonged period. For example, gamete development can fail to complete^19,27–29^, fewer polyps may be fertile within a colony^30,31^, polyps may produce fewer gametes^19,29–32^, fertilisation success may decline^33,34^, or larval development may be abnormal^34,35^. In some cases, coral reproduction has required 4–5 years to recover and return to pre-bleaching levels^19,29^.

Coral bleaching is a clear visual indicator of major environmental stress, but the absence of bleaching does not necessarily preclude stress. Indeed, lower levels of stress can negatively impact the health of corals without showing bleaching. Corals exposed to thermal stress below bleaching thresholds showed changes in the expression of possible stress indicator genes^36,37^, reduction in skeletal growth and impaired recovery from injuries^38^, damage to the morphology and physiology of the coral’s algal symbionts^39^, and a shift in the relation between the coral host and its symbionts, at the cost of the coral host’s health^40^.

While thermal stress is viewed as the main cause of coral bleaching, other environmental factors can be involved as well; notably, solar radiation, both in the visible and the ultraviolet wavelengths, can significantly influence the severity of thermally induced coral bleaching^41,42^. Solar radiation in the visible range (photosynthetically active radiation, PAR: 400–700 nm) is vital to photosynthetic organisms such as the algal symbionts that live within the tissue of reef-building corals; but at levels beyond the organism’s thresholds, the photosystem saturates, and harmful reactive oxygen species (ROS) are released, damaging the photosynthetic apparatus^43–45^. When both temperature and PAR light stress are present, two different pathways of cellular damage to the coral symbionts may interact, increasing the likelihood of a breakdown in the coral–symbiont symbiosis (i.e., coral bleaching) and the degree of that breakdown^46–48^. Solar radiation in the ultraviolet range (ultraviolet radiation, UVR: 280–400 nm) is understood to be detrimental to corals, with effects that include DNA damage, diminished metabolism, bleaching, mortality, and oxidative stress^49^. The cellular physiology and pathways involved during UVR-related stress alone are not fully understood but experiments that manipulated UVR alone revealed rapid lethal effects of full UVR exposure in the worst cases, and reduced growth and calcification in the best cases^50,51^. However, it has also been found that many corals that naturally develop in higher UVR conditions develop UVR-protective compounds such as mycosporine-like amino acids (MAAs), which they can transfer to their eggs, yielding larval survival in higher UVR conditions^52,53^. Nonetheless, when both temperature and UVR stress are present, effects can be additive, causing a stronger response than would be expected by each stressor alone^54,55^.

In the central Pacific archipelago of Hawaiʻi, two major bleaching events occurred in the summers of 2014 and 2015, resulting in widespread mortality across coral assemblages^56,57^. In Kāneʻohe Bay, on the island of Oʻahu, bleaching was extensive but survival rates were high^58^. Nevertheless, reproductive physiology work conducted in Kāneʻohe Bay before, during and after the bleaching events, revealed that sperm motility in at least two coral species dropped nearly by half following the first bleaching event^34^ and remained depressed for the following four years^34,52^. This suggests that the environmental stress that led to extensive coral bleaching in 2014 and 2015 may have caused long-term physiological damage, or perhaps some environmental stress remained, although at sub-bleaching levels.

Here, we investigate the impacts of thermal and solar radiation stress on the biology of yearly coral reproductive events in an experimental system. Specifically, we examine both the individual and combined effects of temperature, ultraviolet radiation (UVR), and photosynthetically active radiation (PAR) in altering reproductive characteristics in the Hawaiian mushroom coral *Lobactis scutaria*, a sequential hermaphroditic species that spawns in the late afternoon, in the days following the full moon, during the Hawaiian summer^34,59^. Environmental factors such as temperature and light are generally interconnected on the reef, and it can be challenging to distinguish the different effects from each other. Therefore, we conducted these experiments in large flow-through aquaria and controlled the temperature, UVR, and PAR levels for 9 months prior to spawning, covering the entire gametogenesis cycle^60^ and presumably altering the early reproductive characteristics of the coral. We report effects on the timing of spawning at the month, day, and time-of-day levels. We report fertilisation success, sperm characteristics such as motility, duration of motility, and early apoptotic signals, and egg volume. Finally, we report changes in coral pigmentation that would indicate early stress levels on the coral holobiont, as well as changes in coral growth throughout the experiment.

## Methods

### Coral collection and husbandry

All experiments were conducted on the scleractinian coral *Lobactis scutaria*, a sequential hermaphroditic, free-living solitary coral from the family Fungiidae, commonly found in Kāneʻohe Bay, Oʻahu, Hawaiʻi^59,61^. Spawning dates of *L. scutaria* in the region are highly reliable with spawning occurring monthly from June to September, 1–4 days after the full moon, in the late afternoon between 17:00 and 19:00^34,59,62^. Two hundred *L. scutaria* individuals were collected on snorkel in Kāneʻohe Bay, Hawaiʻi, in 2015 and 2017 (2015: n = 40; 2017: n = 160), from a depth of 1–5 m, under permits to the Hawaiʻi Institute of Marine Biology # SAP 2015-17, SAP 2016-69, and SAP 2018-03, from the State of Hawaiʻi Department of Land & Natural Resources. The individuals were tagged and kept in captivity in lightly shaded flow-through outdoor aquaria at the Hawaiʻi Institute of Marine Biology on Moku o Loʻe. All aquaria, before and during the experiment, contained yellow tangs (*Zebrasoma flavescens*) and threadfin butterflyfish (*Chaetodon auriga*) to control algae levels and to prevent pest outbreaks. Their diet was supplemented with nori seaweed and brine shrimp three times a week. Grazing fish were maintained under the supervision of the University of Hawaiʻi Office of Research Compliance with the Animal Care and Use Committee protocol # 14-1884-3.

### Experimental setup

**Figure 1:**
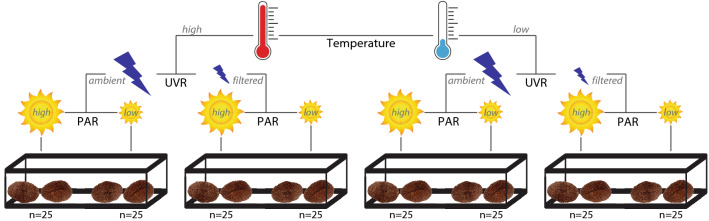
Summary of experimental conditions and number of *L. scutaria* individuals per treatment. Two hundred individuals were divided into eight treatments, each exposed to one of two levels of temperature, ultraviolet radiation (UVR), and photosynthetically active radiation (PAR). The high temperature regimen aims to follow modern temperature records from 2014 to 2019 (includes two bleaching events) and the low temperature regimen aims to follow historical temperatures from 2008–2013. Filtered UVR is 98% filtered. High PAR is 5% shaded and low PAR is 35% shaded.

In late December 2017, prior to the onset of the 9-month-long gametogenesis cycle in *L. scutaria*^60^, all individuals were randomly assigned to one of eight treatment groups with 25 individual mushroom corals in each treatment (Fig. 1). We ensured that there was no difference in average weight and in average colour brightness among the treatment groups prior to starting the experiment (buoyant weight, one-way ANOVA, p > 0.05; colour brightness, one-way ANOVA, p > 0.05). The experimental factors were temperature (2 levels: historical or modern, with modern temperature set ~ 2 °C higher than historical temperature in the summer months only, from mid-May to September), ultraviolet radiation (UVR) (2 levels: ambient or 98% filtered), and photosynthetically active radiation (PAR) (2 levels: high (~ 5% shaded) or low (~ 35% shaded)). The eight treatments were spread into four large 850-L outdoor tanks, with each tank containing two PAR levels. Within a tank, the two groups under different PAR treatments were separated by a 20-cm gap, to account for solar movement and ensure that all corals remained in their PAR treatment at any given time of day. All tanks were supplied by a continuous flow of ambient, warmed or chilled sand-filtered seawater, and were exposed to natural levels of sunshine and moonlight minus the UVR and PAR experimental filtering used in this experiment. Water temperature was not adjusted from December to mid-May, after which it was either warmed or chilled until October. Throughout the experiment, the temperature and the PAR levels were monitored on a bi-weekly basis to verify that the two levels of both factors were constant in each experimental treatment.

**Figure 2:**
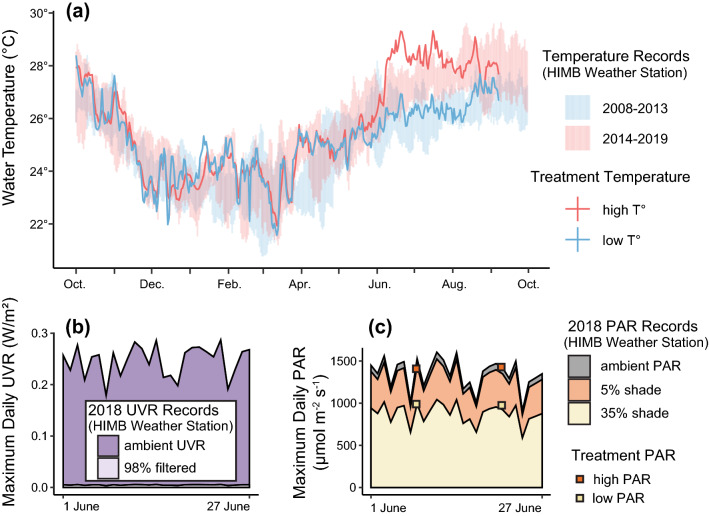
(**a**) Seawater temperatures in the high and low temperature treatments (solid lines) approximately following modern temperature records (2014–2019, includes two bleaching events) and historical temperature records (2008–2013) from the Moku o Loʻe weather station (lighter coloured ranges). (**b**) Ultraviolet ranges in the ambient UVR and 98% blocked UVR treatments visualised here for the month of June 2018, based on records from the Moku o Loʻe weather station. (**c**) Photosynthetically active radiation measurements for the month of June 2018 in the high and low PAR treatments (solid boxes), representing 5% and 35% shading of ambient PAR levels, respectively. Upper line (in grey) represents ambient PAR levels recorded at the Moku o Loʻe weather station. The light orange and light yellow lines are calculated as 5% and 35% less than ambient levels, matching the shading levels in our PAR treatments.

Seawater temperatures in the Hawaiian Islands have gradually been increasing in the past decades^56,63–66^ and in Kāneʻohe Bay, since the 2014 and 2015 bleaching events, they have remained higher than usual^67^. Therefore, we aimed for our two temperature treatments to follow historical (lower) and modern (higher) temperature trends. The temperature treatments were initiated in mid-May, after which the higher temperature was set ~ 2 °C warmer than the lower temperature. To visualise the overall temperature curves, we plotted our experimental temperature data against two sets of water temperature records from Kāneʻohe Bay: 2008–2013 (no bleaching reported in this period) and 2014–2019 (coral bleaching reported in 2014 and 2015) (Fig. 2a). These temperature records were retrieved from an automated weather station located on Moku o Lo‘e at the Hawai‘i Institute of Marine Biology (HIMB) (http://www.pacioos.hawaii.edu/weather/obs-mokuoloe/), which collects hourly temperature data at 1 m depth. Experimental water temperatures were controlled by a chilled water system and aquarium heaters when needed, and were monitored throughout the experiments with temperature loggers collecting hourly data (Onset, HOBO). A daily average was extracted for all temperature data and the maxima and minima temperatures of each 5-year range of temperature data were used to visualise the temperature range during each period, in comparison with our treatment temperatures.

UVR can cause substantial damage to corals^49,54^ and therefore we wanted to test whether blocking UV radiation could prevent damage to the corals’ reproductive physiology. Half of the treatments were exposed to ambient UVR conditions and UVR was reduced by 98% in the other half, using ultraviolet-blocking acrylic sheets (MinPlastics, Honolulu, OP-3 acrylic sheet: blocks ~ 98% UVR at 200–400 nm with partial irradiance (~ 50%) blocked in the visible range at 400–430 nm; Supplementary Fig. S1). To visualise the UVR ranges for both treatments, UVR data were retrieved for the month of June 2018 from HIMB’s weather station (http://www.pacioos.hawaii.edu/weather/obs-mokuoloe/), matching UVR conditions for the ambient UVR treatment, and for the UVR-shielded treatment UVR data were calculated as 2% of the ambient treatment (Fig. 2b).

PAR is a vital environmental input for corals that associate with photosynthetic organisms, as is true of most reef-building corals; but in excess it can cause damage to the algal symbionts’ photosystem apparatus and precipitate coral bleaching^68–73^. The two chosen PAR levels in the PAR treatments represented reductions from natural surface conditions (high PAR: ~ 5% PAR reduction, and low PAR: ~ 35% PAR reduction, determined with an underwater quantum flux reader, model MQ-210, Apogee) and corresponding to conditions encountered on shallow reef flats/edges (~ 0.1–1 m) and shallow reef slopes (~ 2–3 m), respectively. PAR data records were retrieved for the month of June 2018 from HIMB’s weather station (http://www.pacioos.hawaii.edu/weather/obs-mokuoloe/) and plotted against our bi-weekly PAR measurements to visualise the PAR ranges for both PAR treatments (Fig. 2c).

### Spawning timing and gamete collection

On the expected days of spawning (1–4 days after the full moons of June, July, and August), all 200 coral individuals were placed in individual 3-L containers at 15:45, shortly before the expected time of spawning, so that gametes could be collected for each individual. All coral individuals were closely monitored for spawning until 19:30, and those that spawned were recorded and their gametes were collected. Sperm was collected by transfer pipette immediately upon release from the mouths of the individual males and sperm samples that were sufficiently abundant (at least 5 ml at a concentration of 10^7^ sperm cells per ml) were used for sperm physiology analyses. Eggs were left to settle in the bottom of the containers and were collected about one hour after release. Sperm physiology and fertilisation experiments were run during the month of August only and egg physiology was run in June, July, and August.

### Spawning synchrony

Spawning synchrony was assessed for each treatment using the Marquis synchrony index adapted for coral reproduction^74,75^. The synchrony index, here calculated at the daily level, takes into account the proportion of corals spawning on each day that spawning was monitored.

$${S}_{M}=\frac{{d}_{1}}{\sum_{t=1}^{t=n}{d}_{t}}\cdot {p}_{1}+\frac{{d}_{2}}{\sum_{t=1}^{t=n}{d}_{t}}\cdot {p}_{2}+\frac{{d}_{3}}{\sum_{t=1}^{t=n}{d}_{t}}\cdot {p}_{3}+\cdots +\frac{{d}_{n}}{\sum_{t=1}^{t=n}{d}_{t}}\cdot {p}_{n}$$

where *t* is the day of spawning monitoring, *n* is the number of days monitored, *d*_*t*_ is the number of corals spawning on day *t*, *p*_*t*_ is the proportion of corals that spawned on day *t*, and $\sum_{t=1}^{t=n}{d}_{t}$ is the total cumulative number of corals that spawned during the period studied. The Marquis index of synchrony ranges from 0 (spawning spread evenly across the spawning days) to 1 (all corals spawned synchronously on the same days) and can tolerate some missed days of spawning activity, as long as these days only involve a low number of individuals and the peak of spawning (≥ 50% cumulative spawning) has been captured in each month^75^. Given that the spawning synchrony index is focused on the proportion of corals spawning each day, no attempt was made to compensate for corals that spawned more than once during the spawning period.

### Fertilisation success

Spawned eggs and sperm from each treatment were used in fertilisation experiments to assess the effects of temperature, UVR, and PAR on fertilisation success. In fertilisation experiments, eggs from 4 to 10 female fungiids per treatment were fertilised by a sperm pool of 1–6 male fungiids (number dependent on availability each night) from the same treatments following Hagedorn and co-authors^76^. Thirty to 150 eggs from each female were placed in individual scintillation vials containing 5 ml filtered seawater (FSW) and sperm from the sperm pool of their treatment was added (final sperm concentration: 1 × 10^6^ cells/ml). After an hour incubation period to allow for fertilisation to occur, the number of eggs was counted under a dissecting microscope to determine the total number of eggs in each vial and the egg–sperm bath was diluted threefold. Fertilisation success was assessed ~ 12 h later by counting the fertilised eggs under a dissecting microscope. Unfertilised eggs had dissociated by that time. Each fertilisation trial was paired with a control that was run similarly to the fertilisation trial but without sperm, to monitor for accidental fertilisation.

### Sperm physiology

Sperm motility was assessed within five minutes after being collected, using computer-assisted sperm analysis (CASA; Hamilton Thorne, Ceros II System, Olympus BX41 with a 10× objective and green filter) system, following Zuchowicz and co-authors^77^. Motile sperm was differentiated from non-motile sperm using a movement threshold of 0.8, with sperm heads moving > 80% of their head diameter characterised as motile. For each sperm sample, a minimum of five video fields and 200 sperm cells were captured to determine total sperm motility. Sperm motility is often driven by sperm mitochondrial membrane potential^78^ and therefore, to test whether this was the case here, a portion of the sperm was then tested for its mitochondrial function using a JC-1 stain assay (Accuri JC-1 Mitochondrial Potential Assay Kit KR310) and a flow cytometer (BD Accuri C6 Plus Flow Cytometer).

### Egg physiology

The collected eggs were brought to the laboratory and imaged with a Science Supply camera mounted on an Olympus BX41 microscope using a 20× objective and a 0.5× C mount. One to 28 eggs were imaged from each individual. The images were analysed with ImageJ software (U.S. National Institutes of Health, version 1.52 g) to obtain visible egg surface area, using the “Analyse particles” tool. The egg surface area was converted to egg volume, assuming sphericity. Egg volume followed a bimodal distribution due to the presence, in some corals, of very small eggs (Supplementary Fig. S2). A lower volume threshold was therefore set at 0.0007 mm^3^ to remove the smaller egg population from the analysis. Statistical analyses were therefore run only on the larger egg population (0.0007–0.0030 mm^3^, with 1–25 imaged eggs per individual, Supplementary Fig. S3), and the egg volume averaged per individual.

### Coral growth

To examine differences in coral growth throughout the experiment, buoyant weight measurements^79^ were taken at the beginning of the experiment (December 2017) and again at the end of the experiment (September 2018). Buoyant weights were converted to dry weights following Jokiel and co-authors^79^. The difference in dry weights was calculated for each coral and divided by the total number of days to find the average growth per day.

### Bleaching/paling response

While our experimental design was not aimed at causing a full bleaching response in our corals, we expected the high temperature, high UVR and high PAR treatments to cause sufficient stress to induce some loss in symbionts, resulting in some visible paling of the coral hosts. Photographic images of each coral were taken at the beginning (Dec 2017) and at the end (Sep 2018) of the experiment, using an Olympus TG-6 compact camera mounted on a frame in a dark chamber with fixed water depth, fixed lighting (Dolan-Jenner MI-150 light source), and fixed camera settings to maximise uniform capture conditions. The images were converted to greyscale for analysis in ImageJ, which calculated the average brightness for each coral (black = 0, white = 255). Brightness values were compared between the beginning and the end of the experiment, yielding a percent increase in colour brightness as indicator of coral bleaching.

### Statistical analyses

To fulfil normality assumptions, arcsin and square-root transformations were used to normalise percentage data and measurement data, respectively. Mixed-model Analyses of Variance (ANOVA) with temperature, UVR, and PAR as fixed factors were used to test their individual and interactive effects on the timing of spawning and the physiology of the gametes. Genotype was included as random factor in the time of day of spawning analyses. Normality of the residuals was verified by plotting a histogram of the residuals against a normal distribution curve and homoscedasticity was verified by plotting the model residuals against the fitted model. A mixed-model ANOVA with genotype as random factor and month of spawning, temperature, UVR, and PAR as fixed factors was conducted on egg volumes. Sperm physiology data was considered from the month of August only due to different sampling designs in other months. Where relevant, post hoc tests were conducted with least square means pairwise comparisons with Tukey or Sidak adjustments for multiple comparisons. Three sets of two-way ordinal regressions with cumulative link models were used to test for a shift in spawning months among the treatments, with p-values adjusted for multiple comparisons. Proportional odds assumptions were verified using tests of nominal effects and tests of scale effects. We report the log-likelihood, the AIC, and the Condition number of the Hessian for each of the three cumulative link models in the Supplementary Table S3 and present here only the results of the best model, based on the AIC. The relation between sperm motility and percent high mitochondrial membrane potential was assessed with Pearson’s correlation test. Figures were made to show only significant effects with data pooled across non-significant effects. All statistical analyses and their associated figures were conducted using R^80^ and the R packages car^81^, ggplot2^82^, lmerTest^83^, lsmeans^84^, multcomp^85^, multcompView^86^, ordinal^87^, rcompanion^88^, Rmisc^89^, and RVAideMemoire^90^.

## Results

### Spawning timing and synchrony

**Figure 3:**
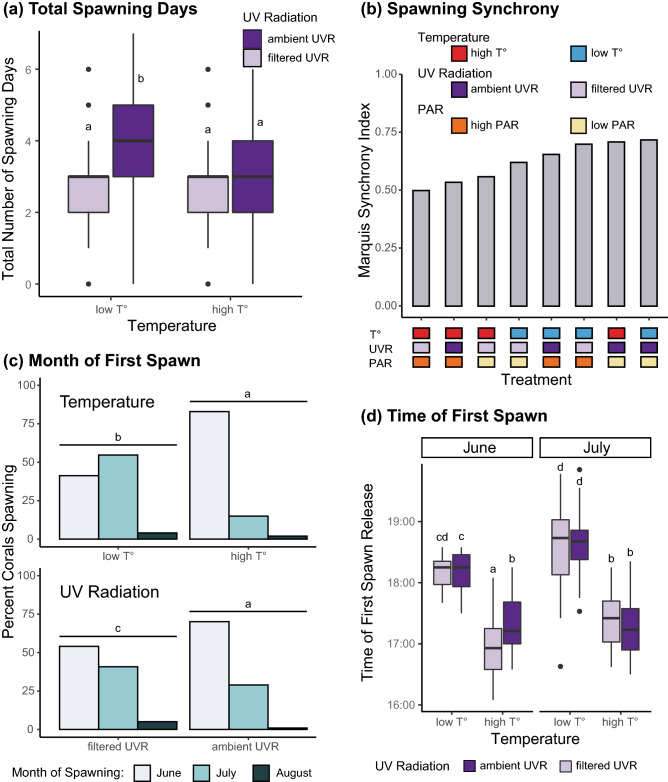
Effects of seawater temperature, PAR, and UV radiation on the timing of coral spawning. (**a**) Both temperature and UVR affected the total number of spawning days that *L. scutaria* spawned in summer 2018. Low temperature and ambient UVR yielded the highest number of spawning days. (**b**) Spawning synchrony was determined with the Marquis Index for each of the eight treatments, and is ranked from low to high synchrony. The lowest synchrony was found at high temperature, blocked UVR and high PAR, and the highest synchrony was found at low temperature, ambient UVR, and low PAR. (**c**) Both temperature and UVR affected the first month of spawning of *L. scutaria*. High temperature and ambient UVR both independently yielded a shift in the initial month of spawning, increasing the percentage of corals starting their spawning period in June rather than July. (**d**) Both temperature and the spawning month had direct effects on the time of day that *L.scutaria* spawned. High temperatures caused corals to spawn earlier than those at low temperatures and spawning occurred later in July in comparison to June. UVR significantly interacted with the month of spawning but did not have a direct effect on the time of spawning. Treatments that share the same letter are not significantly different from each other.

Spawning occurred in June, July, and August, in the week following the full moon. The number of spawning days per individual ranged from 0 to 7, with an average of 3.19 ± 0.11 days (mean ± standard error) (Supplementary Table S1). However, the number of days during which each coral spawned was affected by both temperature and UVR (mixed-model ANOVA, F_(1,188)_ = 13.9, p = 3 × 10^–4^ and F_(1,188)_ = 22.4, p = 4 × 10^–6^, respectively, Fig. 3a, Supplementary Table S2). Higher temperature regimens caused a drop in the number of spawning days compared to lower temperatures, and blocking 98% UVR caused a similar drop in the number of spawning days (Fig. 3a). Additionally, temperature and UVR had a significant interaction effect (mixed-model ANOVA, F_(1,188)_ = 6.6, p = 0.011, Fig. 3a, Supplementary Table S2), with lower temperatures and ambient UVR causing a significantly higher number of spawning days than any other combination (post hoc test: least square means with Tukey correction).

Within each treatment, spawning synchrony determined by the Marquis index^74,75^ ranged from 0.50 to 0.72 (scale: 0 to 1). The lowest synchrony occurred at higher temperature regimens with blocked UVR and high PAR, and the highest synchrony was found at lower temperatures, ambient UVR and low PAR (Fig. 3b). Overall, synchrony appeared to be the most affected when temperature and PAR were on the higher end, and when UVR was filtered out.

Overall, 62% of fungiid corals first spawned in the month of June in 2018 (Supplementary Table S1). However, the onset of spawning at the month level was affected by both temperature (two-way ordinal regression, χ^2^ = 38.2, p = 2 × 10^–9^) and UVR (two-way ordinal regression, χ^2^ = 6.8, p = 0.028), with higher temperatures and ambient UVR increasing the proportion of corals that first spawned in the month of June, and lower temperatures and blocked UVR increasing the number of corals that shifted the onset of spawning to the month of July (Fig. 3c; Supplementary Table S3).

Throughout the 2018 summer months, spawning was observed between 16:00 and 18:30 (Supplementary Table S1). The time of day that spawning started was affected both by the month (mixed-model ANOVA, F_(1,285)_ = 36.9, p = 4 × 10^–9^) and by temperature (mixed-model ANOVA, F_(1,199)_ = 364.1, p < 2 × 10^–16^) (Supplementary Table S4). Spawning occurred on average 40 min later in July than in June, and corals at higher temperatures spawned on average 80 min earlier than ones at lower temperatures (Fig. 3d). Interactive effects were also noted between UVR and month (mixed-model ANOVA, F_(1,285)_ = 3.9, p = 0.050), with UVR causing a difference in spawning time in June but not in July, and among temperature, UVR and month (mixed-model ANOVA, F_(1,285)_ = 7.7, p = 0.006) (Fig. 3d; Supplementary Table S4). Data from the month of August were not included in the statistical analysis. In August, exceptional conditions (Hurricane Lane^91^) forced us to evacuate all corals out of their treatments and into a single solid emergency aquarium that was less likely to be destroyed in case of a direct hit by the hurricane, for a period of three days. After three days, the corals were returned to their treatments and monitored that same day for spawning. No significant month effect was observed between July and August (corals spawned on average only 4 min later). However, the delay between temperature treatments had disappeared (under high temperatures, spawning started only 4 min earlier than under lower temperatures).

### Fertilisation success

**Figure 4:**
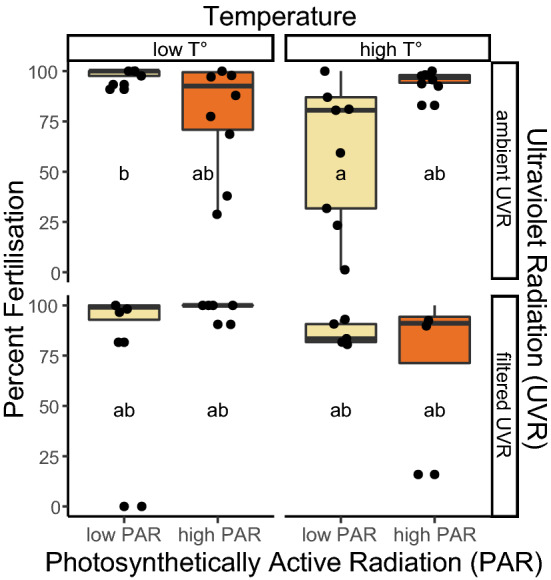
Fertilisation success was directly impacted only by temperature, with a reduction in percent fertilisation at high temperatures. UVR and PAR had significant interactive effects with temperature but did not show any direct effect on fertilisation. Treatments that share the same letter are not significantly different from each other.

Fertilisation success within treatments was overall 85 ± 3% (mean ± standard error) (Supplementary Table S5). Higher temperatures decreased fertilisation success (mixed-model ANOVA, F_(1,52)_ = 4.8, p = 0.033) to 80% on average in the warmer treatments. Fertilisation success was also impacted by combined effects of temperature and PAR (mixed-model ANOVA, F_(1,52)_ = 5.0, p = 0.029), and by the combined effects of temperature, PAR and UVR (mixed-model ANOVA, F_(1,52)_ = 6.9, p = 0.011, Fig. 4; Supplementary Table S6).

### Sperm physiology

**Figure 5:**
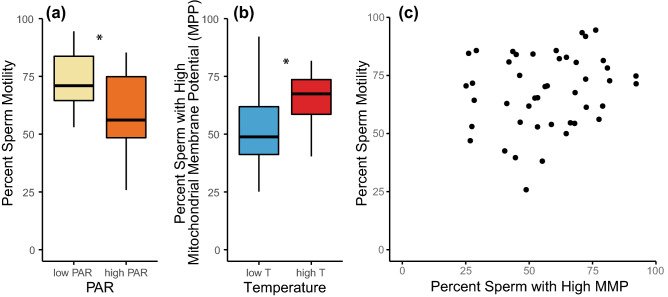
Sperm physiology was affected by PAR and temperature. Sperm motility (**a**) was sensitive to PAR and was lower at ambient PAR levels. Mitochondrial membrane potential (**b**) was sensitive to temperature and was lower at low temperature levels. A star * means that the treatments were significantly different from each other. (**c**) There was no significant correlation between percent sperm with high mitochondrial membrane potential and percent sperm motility (p = 0.198).

Sperm motility, assessed by CASA, ranged from 26 to 95% (Supplementary Table S7) and was significantly affected by PAR (mixed-model ANOVA, F_(1,37)_ = 1841, p = 0.08), with the low PAR treatment increasing sperm motility from 61% (average motility in the high PAR treatment) to 73% (Fig. 5a; Supplementary Table S8). Differences in sperm motility can often be explained by changes in sperm mitochondrial membrane potential (MMP). High MMP is generally expected but low MMP would reveal some damage to the sperm. The percentage of corals with high sperm MMP was highly variable, ranging from 25 to 92% (Supplementary Table S7). Unlike sperm motility, MMP was affected by temperature (mixed-model ANOVA, F_(1,37)_ = 8.9, p = 0.005) with a higher proportion of corals with high MMP at higher temperatures in comparison to historical temperatures, respectively 66% and 51% on average (Fig. 5b; Supplementary Table S9). Sperm motility and high mitochondrial membrane potential were not significantly correlated (Fig. 5c, Pearson’s correlation, r = 0.19, t_(43)_ = 1.3, p = 0.198).

### Egg physiology

**Figure 6:**
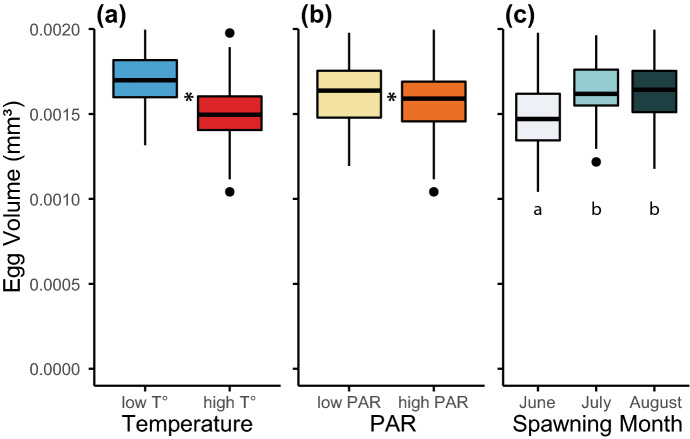
The size of the eggs that were released each month was affected by temperature (**a**), PAR (**b**), and the month that the eggs were released (**c**). Higher temperatures yielded a smaller egg size, as did high PAR levels. In June, the eggs were smaller than in July and August. A star * means that the treatments were significantly different from each other. Treatments that share the same letter are not significantly different from each other.

The size of the eggs released by *L. scutaria* was significantly affected by temperature, PAR, and the month of spawning (mixed-model ANOVA: F_(1,112)_ = 46.4, p = 5 × 10^–10^; F_(1,119)_ = 4.4, p = 0.038; F_(2,109)_ = 7.1, p = 0.001, respectively; Supplementary Table S11). Higher temperature reduced egg size by 14%, high PAR reduced egg size by 3%, and in June, eggs were 10% smaller than in July and August (Fig. 6; Supplementary Table S10).

### Coral growth

**Figure 7:**
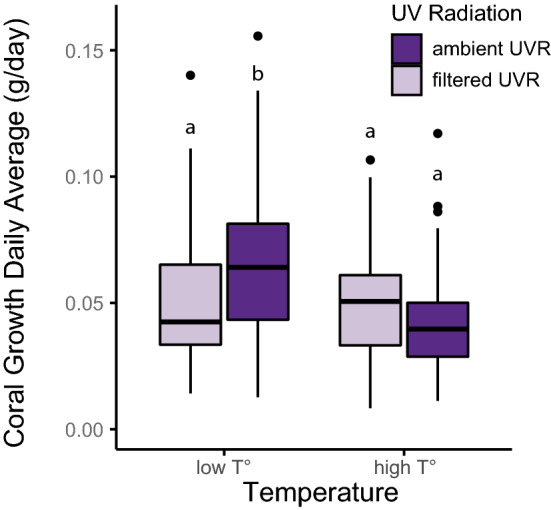
Coral growth was affected by temperature, with reduced growth in high temperature regimens. UVR interacted with temperature but did not have a direct effect on growth. Treatments that share the same letter are not significantly different from each other.

A significant reduction in growth was observed in the higher temperature treatments (mixed-model ANOVA, F_(1,186)_ = 11.2, p = 0.001; Fig. 7; Tables S12–13). Temperature and UVR had a significant interactive effect on growth with UVR causing a significant difference at low temperatures but not at high temperatures (mixed-model ANOVA, F_(1,186)_ = 8.6, p = 0.004; Fig. 7; Supplementary Table S13). The highest growth rates were found at low temperature and ambient UVR treatments.

### Bleaching/paling response

**Figure 8:**
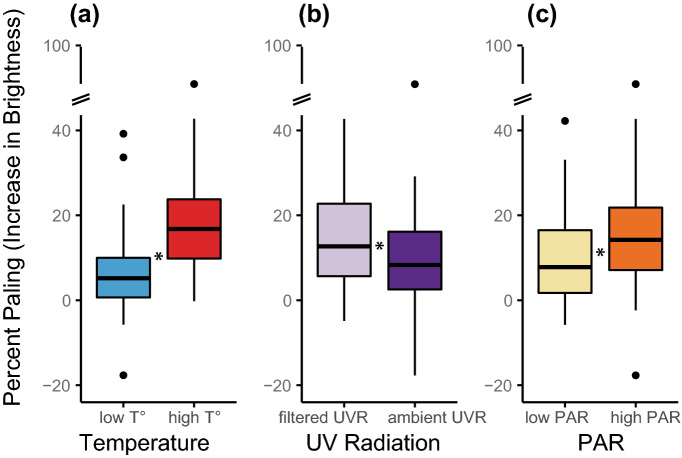
Some degree of coral paling occurred in all treatments between December 2017 and September 2018. However, corals paled significantly more at high temperature (**a**), when UVR was filtered out (**b**), and at high PAR levels (**c**). A star * means that the treatments were significantly different from each other.

Considering all treatments together, corals paled by 12 ± 1% (mean ± standard error) between December 2017 and September 2018 (Supplementary Table S12). Nevertheless, the change in coral pigmentation was affected by temperature, UVR, and PAR (mixed-model ANOVA: F_(1,191)_ = 93.9, p < 2 × 10^–16^; F_(1,191)_ = 18.4, p = 3 × 10^–5^; F_(1,191)_ = 23.5, p = 3 × 10^–6^, respectively; Supplementary Table S14). Higher temperature caused 18% paling, high PAR caused 15% paling, and blocked UVR caused 14% paling (Fig. 8).

## Discussion

Temperature, ultraviolet radiation (UVR) and photosynthetically active radiation (PAR) all played significant but different roles in affecting the reproductive biology of the coral *L. scutaria*. The present study showed that timing of spawning was affected by higher temperature and blocked UVR but not by PAR. Fertilisation success was affected by higher temperature, and sperm and egg physiology were affected both by temperature and PAR. Growth was affected by temperature, and corals paled more at the higher levels of temperature and PAR and when UVR was filtered out. The interactions of thermal stress with light stress both at the PAR level and UVR level are clearly complex, but understanding the individual role of each factor, and understanding how they interact, provides vital information to better understand how to manage these stressors in the changing environment that we are expecting in the coming decades.

Sessile animals such as corals rely on the synchronous release of gametes from different genotypes to cross-fertilise and produce viable offspring^92,93^. We found that the number of opportunities for cross-fertilisation (i.e. total number of spawning days) dropped with warmer temperatures and when UVR was blocked. Similarly, we found that spawning synchrony, determined with the Marquis synchrony index, also dropped in a warmer temperature regimen and when UVR was blocked. Higher synchrony occurred in conditions that yielded a higher number of spawning days, and synchrony dropped when conditions yielded a drop in number of spawning days. This relationship can happen when the number and proportion of corals involved in spawning is high on each day^75^. Indeed, the higher the synchrony and the more opportunities for a high proportion of the coral population to spawn on the same day, the higher the chances of reproductive success on the reef^94^. The opposite trend (i.e. high number of spawning days related to low synchrony) was recently suggested to occur in coral communities in Eilat, northern Red Sea^95^, but the authors did not quantify synchrony directly and did not analyse comparable datasets, so further work would be needed to confirm the trend^96^. In our case, in Hawai‘i, we found that an increase in temperature yielded fewer spawning days and a drop in spawning synchrony. Oceans around the globe have been warming steadily since the 1970s^97,98^ and are likely to continue warming in the next decades^99^, potentially affecting spawning synchrony throughout coral populations globally in the coming years. We found that spawning synchrony was also lower when UVR was blocked. Corals are believed to follow lunar cues to determine the days of spawning, which involves detecting low levels of blue light, which is adjacent to the ultraviolet spectrum^100–102^. Blocking UVR in half of our treatments may have affected those corals’ ability to detect the lunar cues in the blue range. It is also possible that the lunar cues needed for spawning synchrony may overlap with the longer wavelengths of the ultraviolet range.

Both warmer temperature regimens and ambient UVR caused a proportion of corals to start spawning a month earlier. By shifting the onset of spawning to an earlier month, these corals can spawn in better thermal conditions, showing potential for a favourable adaptation to warming conditions. A similar shift in the month of spawning has been reported in *Acropora japonica* (but not in *A. hyacinthus*), in *Favites pentagona*, and in *Platygyra contorta* in Japan, where spawning occurred a month earlier when seawater temperatures were higher than average during the last three months of gametogenesis^103^.

In addition to a month shift in the onset of spawning, we found that warmer temperatures caused a shift in the time of day that spawning began, with corals exposed to higher temperatures spawning 80 min earlier on average. Most scleractinian corals spawn at night, in the hours following sunset, but *L. scutaria* spawns in the late afternoon, presumably not using the sunset as a proximate cue for the time of spawning as night-spawning corals do^104–106^. While adapting to warming temperature by advancing the onset of spawning to an earlier month is advantageous in avoiding hotter water temperatures, spawning earlier in the afternoon will cause gametes to be released at a time of the day when the water is warmer and insolation is stronger. Hotter-than-normal temperatures at the time of gamete release can reduce fertilisation success, cause abnormalities in developing larvae, and reduce larval survival^107,108^. In addition, stronger insolation can lead to DNA damage to the gametes and developing larvae due to stronger ultraviolet radiation^49,109,110^. While this effect is concerning, we found that the shift in spawning to earlier in the afternoon caused by higher water temperature can be reset in just three days, as we accidentally found out when we had to evacuate all corals to a safer tank due to Hurricane Lane in August 2018. While the exact mechanisms by which the fungiid corals synchronise their spawning to the same time of day remain unknown, we found that water temperature played a significant role, either as a direct cue, or through disrupting the mechanisms responsible for determining the timing.

Fertilisation success is highly dependent on a sufficiently high concentration of motile sperm^33,77^. Once above a minimum threshold of 5 × 10^3^ sperm cells per egg for the coral *L. scutaria*^77^, fertilisation is generally high, which likely explains why we obtained high fertilisation success in all treatments. Higher temperature caused a drop in fertilisation success with the lowest fertilisation rates observed at warmer temperature, low PAR and ambient UVR. A few studies have investigated the effect of short-term increased temperature during the stages of fertilisation success and larval development in corals and have found that an increase in temperature during these stages tended to accelerate the larval development^107^ but did not necessarily cause a drop in fertilisation success, except in *Acropora millepora*^107^. In the coral *A. tenuis*, the sperm concentration threshold necessary for successful fertilisation was found to be possibly higher at increased temperatures, requiring approximately a six to eightfold higher sperm concentration at 3 °C above ambient temperatures in the coral *A. tenuis*^111^. However, the authors did not evaluate sperm motility, so the need for increased sperm concentration could simply have been to compensate for lower sperm motility at higher temperature to obtain similar fertilisation success rates. Following bleaching events, drops in fertilisation have been apparent in several coral species^33,34^, but many confounding effects other than temperature could have been involved. In the present study, we noted a temperature effect but we would suggest further studies using lower concentrations of motile sperm to identify the limits of fertilisation success under different levels of environmental stress.

Unlike fertilisation success, sperm motility dropped at high PAR and was not affected by temperature. The absence of a temperature effect suggests that although some sperm motility is important for fertilisation success, here, other factors may be involved. For example, we found the strongest effect on egg volume to be temperature, with a drop in egg size at warmer temperatures. The smaller eggs might have been the cause of the reduced fertilisation success in the higher temperature treatments. Sperm motility has been overall low since two consecutive bleaching events in Hawaiʻi in 2014 and 2015^34^. Loss of sperm motility can be associated with a loss in sperm mitochondrial membrane potential (MMP) in humans^112,113^ and bovines^114^, but also in marine invertebrates such as sea urchins^78,115^ and in some coral species^52^. Indeed, a high proportion of high MMP reflects the process of electron transport and oxidative phosphorylation, the driving force behind ATP production and therefore sperm motility. However, this relationship was not found in sperm from marine ascidians or mussels; loss of sperm motility was instead correlated to reactive oxygen species levels and plasma membrane lipid peroxidation^114^. Similarly, in this study, we found no relation between MMP and sperm motility in the coral *L. scutaria*. Here, the cause of the loss of sperm motility is likely elsewhere. That sperm motility was diminished at high PAR suggests that the stress that drove sperm motility here may have occurred, to some extent, at the level of the algal symbiont, the photosynthetic partner in the symbiosis, during spermatogenesis. Several studies report negative effects from UVR or combined UVR and PAR on the sperm motility of corals^116^ and other marine invertebrates^117,118^, but few from PAR alone. In a study comparing DNA damage, a sign of advanced cell apoptosis, in coral larvae with and without symbionts when exposed to direct insolation (high PAR and high UVR were coupled), DNA damage was higher in larvae with symbionts than in larvae without symbionts, indicating that the symbionts were the source of oxidative stress^119^. To understand to what extent oxidative stress in the symbionts led to a decrease in sperm motility, further work is needed, potentially testing gene expression regulatory mechanisms, alongside high-resolution imaging to identify any morphological anomalies. Testing the direct effects of reactive oxygen species, the toxic compound released during photo-oxidative stress, on sperm cells, during their development or after their release, might also provide valuable answers.

The volume of the eggs released by female *L. scutaria* individuals was reduced at warmer temperatures, but it was also lower at higher PAR and was lower in the first month of spawning (i.e. June). A similar temperature effect was found in the coral *Acropora digitifera* in Okinawa when exposed to temperature regimens 2 °C above ambient temperatures over several weeks prior to spawning^120^. Eggs are mostly composed of lipids, which will act as energy reserves for the developing larva. To build up enough lipids to produce larger eggs, sufficient resources need to be available. These are provided by the photosynthetic symbionts as part of their symbiotic relationship with their coral host, which relies on the symbionts being healthy and abundant. In this study we found that both temperature and PAR caused corals to pale, which indicates that the symbionts were at the very least less abundant and possibly not able to provide as many nutrients to their coral host as their counterparts in the lower temperature and PAR treatments.

Although warmer temperatures, PAR, and UVR all caused paling of corals, coral growth was affected only by the warmer temperature treatments, which we had adjusted to approximately follow recent temperature observations on reefs in Kāneʻohe Bay (based on data 2014–2019). Previous work in Kāneʻohe Bay has shown that the optimal temperature for coral calcification, including for *L. scutaria*, is 25.9 °C and that coral growth drops when above that threshold^121^. Our results confirm these earlier results and suggest that coral growth may already be dropping throughout Kāneʻohe Bay due to ocean warming. Elsewhere, reductions in coral growth linked with warming ocean temperatures have already been detected in the Red Sea^122^, on the Great Barrier Reef^123^, in the Andaman Sea^124,125^, and in the South China Sea^125^.

The physiological effects of global climate change on coral reproduction are numerous and complex^126^. However, the bottom line is that without robust reproduction, corals may not be able to adapt to changing ocean conditions. We show that warming ocean temperatures and high solar radiation both negatively affect the reproductive physiology of the mushroom coral *L. scutaria*, but they may do so through different cellular pathways. Oceans are predicted to continue warming in the decades to come, which may cause a further reduction in coral spawning synchrony, disrupt the timing of spawning, cause a further drop in fertilisation success and in egg volume, and slow coral growth. To manage warming ocean temperatures will take a global effort and strong political will and action, but that effort is necessary to prevent the loss of coral reefs and the vital ecosystem services that they provide^127^.

While high levels of PAR are likely to continue to affect the reproductive physiology of corals in the coming decades, artificially controlling PAR levels may help reduce damage to sperm and eggs, and mitigate bleaching effects under thermal stress. Shading corals has been tested for a variety of species, showing promising results in reducing coral bleaching and decreasing effects on overall fitness^73^. Our results suggest that it could also prevent damage to coral gametes, thus reducing the risk of reproductive failure. However, such measures would be challenging to apply in a natural environment and may need to be limited to small-scale reef patches and land-based coral nurseries. Manipulating PAR levels should also consider that below a certain threshold, the circadian rhythm of the corals may be disrupted, causing unwanted damage to the corals’ life cycle^128,129^ so shading mitigation measures would need to be undertaken with caution^10,130,131^. Finally, high levels of ultraviolet radiation are known to cause cellular damage in many organisms but lower levels of UVR may play an important functional role. Here, we show that completely blocking UVR can impair lunar cycle–related cues, on which the corals rely for determining the timing of spawning and maintaining spawning synchrony.

The coming decades will be challenging for coral reefs around the world, but among the key steps to conserving reef-building corals will be to ensure that reproduction is maintained and to better understand the factors that may disrupt it. This will require global, annual monitoring of coral reproduction alongside further research on the effects of climate change on coral reproductive physiology and reproduction timing. This study is a first step in that direction but many effects identified here would benefit from more in-depth studies focused on these effects, and more importantly, this study needs to be repeated on other coral species to determine how whole coral reef communities are likely to respond to some of the environmental changes associated with climate change in the coming years. On a longer time-scale, global pressures from climate change will need to be addressed for oceans to return to environmental conditions more suitable for the world’s coral reefs to thrive again.

## Supplementary Information

Supplementary Information.

## Data Availability

The data supporting the findings of this paper are available in the article and Supplementary Information.
