# Supplementary material for: Solar radiation, temperature and the reproductive biology of the coral Lobactis scutaria in a changing climate
Source: Sci Rep. 2023 Jan 5;13:246. doi: 10.1038/s41598-022-27207-6 (PMC9816315; doi:10.1038/s41598-022-27207-6)
Supplement: Supplementary file 1 — Supplementary Information. [file 41598_2022_27207_MOESM1_ESM.pdf]

Supplementary Information

Solar radiation, temperature and the reproductive biology of the coral *Lobactis scutaria* in a changing climate

Jessica Bouwmeester[1,2,*], Jonathan Daly[1,2], Nikolas Zuchowicz[1,2], Claire Lager[1,2], E. Michael Henley[1,2], Mariko Quinn[1,2], Mary Hagedorn[1,2]

[1]Smithsonian Conservation Biology Institute, Front Royal, VA 22630, United States of America
[2]Hawaiʻi Institute of Marine Biology, Kāneʻohe, HI 96744, United States of America

*Correspondence: Jessica Bouwmeester. Email: jess.bouwmeester@gmail.com

This file contains:

I. UVR Filtering Plexiglas Figure
Figures S1

II. Egg Physiology Figures
Figures S2-S3

III. Coral Spawning Timing Tables
Tables S1-S4

IV. Fertilisation Success Tables
Tables S5-S6

V. Sperm and Egg Physiology Tables
Tables S7-S11

VI. Other Physiology Characteristics Tables
Tables S12-S14

I. UVR Filtering Plexiglas Figure

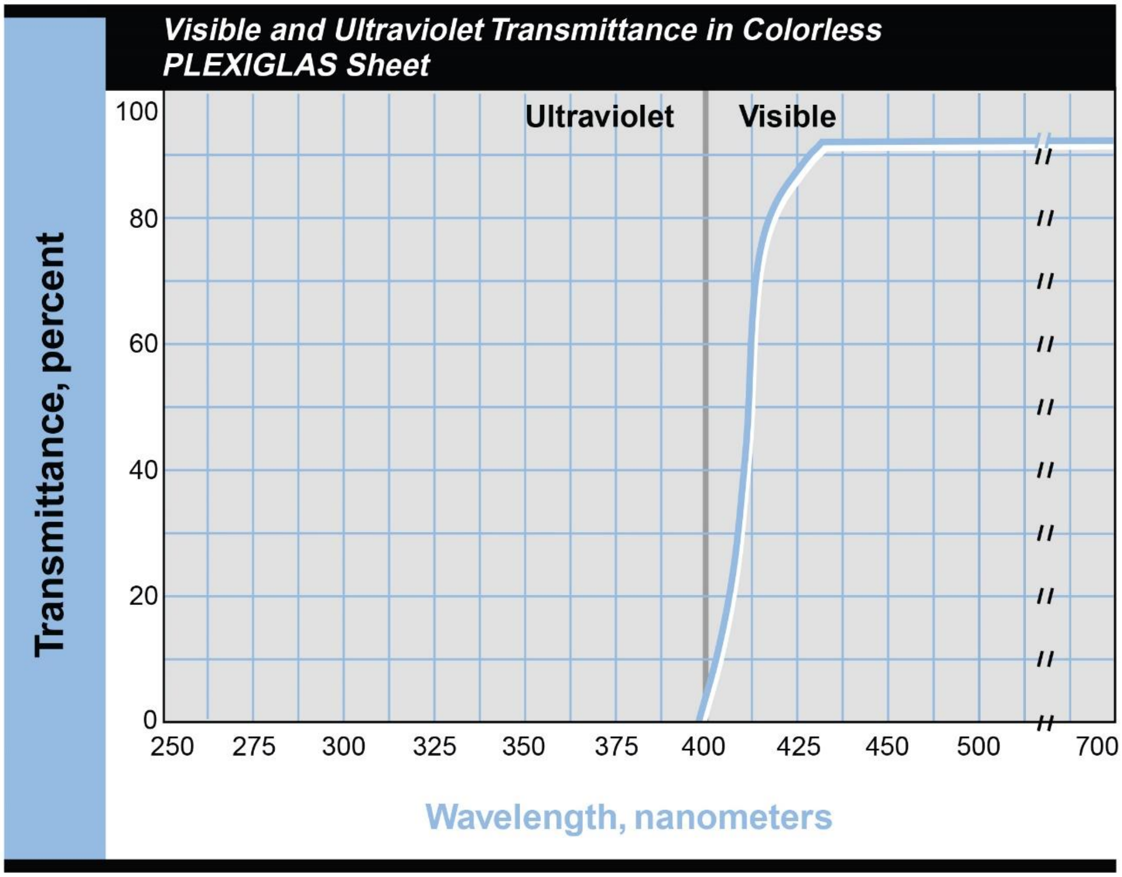

Figure S1: Visible and ultraviolet transmittance of the Plexiglas used in our experiments to filter out ultraviolet radiation. Extracted from: Altuglas International, Arkema Group (2000) Plexiglas Acrylic Sheet, Optical & Transmission Characteristics, 12 pages.

II. Egg Physiology Figures

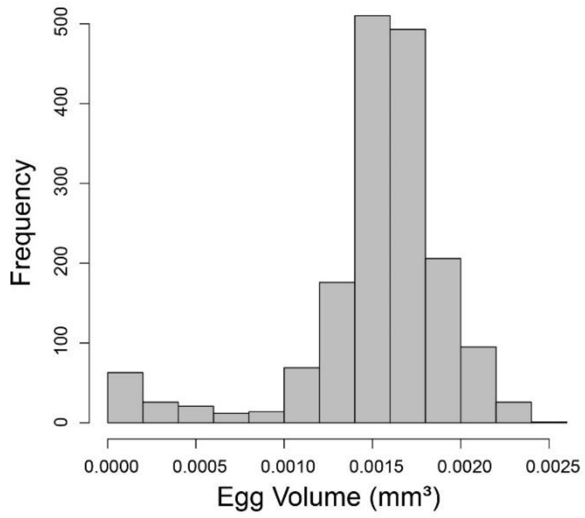

Figure S2: Frequency distribution of the volume of all eggs collected and imaged in June, July, and August 2018. A bimodal distribution is observed so a lower volume threshold was set at 0.0007 mm^3 to remove the smaller population.

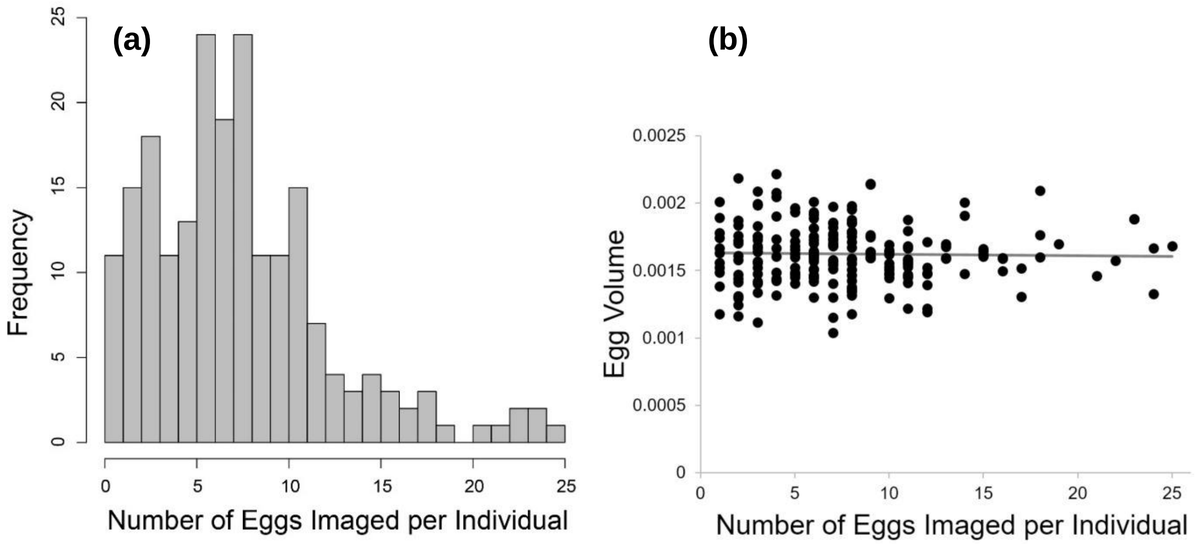

Figure S3: (a) Frequency distribution of the number of eggs imaged and used in the present study's size analysis, after a lower volume population (see Figure S1) was removed from the analysis. **(b)** Relationship between the number of eggs imaged per individual and the average egg volume for each individual, to verify that the egg volume was not biased in cases with a low number of eggs imaged per individual. The trendline indicates that the overall average egg volume does not vary with the number of replicates although higher replicate numbers (>12) appear to yield lower variability.

III. Coral Spawning Timing Tables

Table S1: Timing of spawning per treatment, showing mean values, number of genotypes (N) and the standard error of the mean

	**High Temperature**				**Low Temperature**			
	**High UVR**		**Low UVR**		**High UVR**		**Low UVR**	
	**High PAR**	**Low PAR**	**High PAR**	**Low PAR**	**High PAR**	**Low PAR**	**High PAR**	**Low PAR**
**Number of spawning days**								
Mean	2.60	3.48	2.75	2.48	4.08	4.46	2.64	3.04
N	25	25	24	23	25	24	25	25
Std Error	0.31	0.27	0.30	0.21	0.36	0.24	0.20	0.28
**First month of spawning** (6=June, 7=July, 8=August)								
Mean[a]	6.23	6.12	6.20	6.21	6.37	6.52	6.88	6.75
N	22	24	24	25	24	25	25	24
Std Error	0.09	0.07	0.10	0.10	0.10	0.12	0.12	0.09
**Time of spawn: June**								
Mean	17:20	17:16	17:00	16:51	18:08	18:16	18:08	18:11
N	17	21	19	19	15	14	6	6
Std Error	00:06	00:05	00:05	00:06	00:05	00:04	00:07	00:07
**Time of spawn: July**								
Mean	17:07	17:23	17:22	17:22	18:34	18:43	18:35	18:37
N	18	23	14	18	24	24	22	24
Std Error	00:05	00:05	00:05	00:06	00:04	00:03	00:06	00:08

[a]Mean values calculated only for the purpose of this table. Months were analyzed as categorical variables.

Table S2: Summary of mixed-model ANOVA on the timing of spawning. Effects on the number of days that each individual spawned, with temperature, UVR, and PAR as fixed factors

	**Number of spawning days**				
	df	SS	F	p-value	
Temperature	1, 188	26.26	13.91	3×10^{-4}	***
UVR	1, 188	42.33	22.42	4×10^{-6}	***
PAR	1, 188	6.21	3.29	0.071	
Temperature x UVR	1, 188	12.48	6.61	0.011	*
Temperature x PAR	1, 188	0.08	0.04	0.841	
UVR x PAR	1, 188	3.82	2.02	0.156	
Temperature x UVR x PAR	1, 188	4.21	2.23	0.137	

Table S3: Summary of two-way ordinal regression for cumulative link models on the timing of spawning. Effects on the first month of spawning

	Difference in first month of spawning				
	df	χ^2	p-value	adj. p-value#	
**Factors: Temperature and UVR**					
Temperature	1	38.214	6×10^{-10}	2×10^{-9}	***
UVR	1	6.788	0.009	0.028	*
Temperature x UVR	1	3.817	0.051	0.152	
**Model Summary**					
Log-Likelihood: -125.55					
AIC: 261.11					
Condition number of the Hessian: 68					
**Factors: Temperature and PAR**					
Temperature	1	36.923	1×10^{-9}	4×10^{-9}	***
PAR	1	0.052	0.819	1	
Temperature x PAR	1	0.318	0.572	1	
**Model Summary**					
Log-Likelihood: -130.67					
AIC: 271.34					
Condition number of the Hessian: 73					
**Factors: UVR and PAR**					
UVR	1	5.469	0.019	0.058	
PAR	1	0.024	0.876	1	
UVR x PAR	1	0.064	0.800	1	
**Model Summary**					
Log-Likelihood: -146.53					
AIC: 303.05					
Condition number of the Hessian: 58					

Bonferroni correction for three repetitions given the two-way ordinal regressions was run three times with different combinations of fixed factors.

Table S4: Summary of mixed-model ANOVA on the timing of spawning, with spawning month, temperature, UVR, and PAR as fixed factors, and genotype as random factor.

	**Time of first spawn**				
	df	SS	F	p-value	
Spawn. month	1, 285	6.38	36.87	4×10^{-9}	***
Temperature	1, 199	63.05	364.07	$< 2 \times 10^{-16}$	***
UVR	1, 199	0.30	1.73	0.189	
PAR	1, 202	0.16	0.89	0.346	
Spawn. month x Temperature	1, 285	0.50	2.89	0.090	
Spawn. month x UVR	1, 285	0.67	3.89	0.050	*
Temperature x UVR	1, 199	0.19	1.09	0.297	
Spawn. month x PAR	1, 287	0.06	0.32	0.571	
Temperature x PAR	1, 202	0.14	0.82	0.366	
UVR x PAR	1, 202	0.19	1.12	0.292	
Spawn. month x Temperature x UVR	1, 285	1.34	7.73	0.006	**
Spawn. month x Temperature x PAR	1, 287	0.26	1.49	0.224	
Spawn. month x UVR x PAR	1, 287	0.06	0.34	0.558	
Temperature x UVR x PAR	1, 202	0.03	0.17	0.679	
Spawn. month x Temperature x UVR x PAR	1, 287	0.02	0.10	0.755	

IV. Fertilisation Success Tables

Table S5: Fertilisation success per treatment, showing mean values, number of genotypes (N) and the standard error of the mean

	**High Temperature**				**Low Temperature**			
	**High UVR**		**Low UVR**		**High UVR**		**Low UVR**	
	**High PAR**	**Low PAR**	**High PAR**	**Low PAR**	**High PAR**	**Low PAR**	**High PAR**	**Low PAR**
**Percent Fertilization**								
Mean	95.44	62.70	74.53	85.90	79.58	97.99	98.11	84.55
N	10	9	4	5	10	9	5	8
Std Error	1.58	11.98	19.67	2.51	8.43	1.15	1.89	12.28

Table S6: Summary of mixed-model ANOVA on fertilisation success with temperature, UVR and PAR as fixed factors. ANOVA performed on arcsin-transformed data.

	**Percent Fertilisation Success**				
	df	SS	F	p-value	
Temperature	1, 52	0.79	4.81	0.033	*
UVR	1, 52	0.006	0.04	0.850	
PAR	1, 52	0.11	0.68	0.415	
Temperature x UVR	1, 52	0.02	0.14	0.714	
Temperature x PAR	1, 52	0.82	5.04	0.029	*
UVR x PAR	1, 52	0.03	0.20	0.654	
Temperature x UVR x PAR	1, 52	1.13	6.92	0.011	*

V. Sperm and Egg Physiology Tables

Table S7: Sperm physiology per treatment, showing mean values, number of genotypes (N) and the standard error of the mean

	High Temperature				Low Temperature			
	High UVR		Low UVR		High UVR		Low UVR	
	High PAR	Low PAR	High PAR	Low PAR	High PAR	Low PAR	High PAR	Low PAR
**Percent Sperm motility**								
Mean[a]	59.64	72.09	68.75	92.60	60.70	72.29	58.54	70.46
N	4	8	2	2	7	9	6	7
Std Error	7.89	4.40	12.65	0.80	8.41	3.59	7.19	4.64
**Percent High Mitochondrial Membrane Potential**								
Mean	64.76	61.61	78.39	71.56	57.16	44.65	44.41	60.86
N	4	8	2	2	7	9	6	7
Std Error	8.85	3.56	0.84	0.68	6.95	6.49	5.91	8.27

Table S8: Summary of mixed-model ANOVA on sperm physiology. Effects on sperm motility

	**Sperm Motility**				
	df	SS	F	p-value	
Temperature	1, 37	216.9	0.94	0.338	
UVR	1, 37	93.8	0.41	0.527	
PAR	1, 37	1841.1	8.00	0.008	**
Temperature x UVR	1, 37	624.3	2.71	0.108	
Temperature x PAR	1, 37	46.7	0.20	0.655	
UVR x PAR	1, 37	31.4	0.14	0.714	
Temperature x UVR x PAR	1, 37	63.2	0.27	0.603	

Table S9: Summary of mixed-model ANOVA on sperm physiology. Effects on the sperm mitochondrial membrane potential, with high mitochondrial membrane potential indicative of healthy cells and low mitochondrial membrane potential indicative of early stages of apoptosis

	**Sperm High Mitochondrial Membrane Potential**				
	df	SS	F	p-value	
Temperature	1, 37	2563	8.91	0.005	**
UVR	1, 37	318	1.11	0.299	
PAR	1, 37	12	0.04	0.840	
Temperature x UVR	1, 37	152	0.53	0.471	
Temperature x PAR	1, 37	4	0.01	0.912	
UVR x PAR	1, 37	949	3.30	0.077	
Temperature x UVR x PAR	1, 37	549	1.91	0.175	

Table S10: Egg size per treatment, showing mean values, number of genotypes (N) and the standard error of the mean

	High Temperature				Low Temperature			
	High UVR		Low UVR		High UVR		Low UVR	
	High PAR	Low PAR	High PAR	Low PAR	High PAR	Low PAR	High PAR	Low PAR
**Egg Volume: June** (mm^3)								
Mean	0.00123	0.00151	0.00135	0.00148	0.00168	0.00176	0.00157	0.00171
N	3	8	16	13	5	5	2	3
Std Error	0.00009	0.00005	0.00004	0.00005	0.00009	0.00009	0.00003	0.00010
**Egg Volume: July** (mm^3)								
Mean	0.00159	0.00153	0.00152	0.00152	0.00174	0.00181	0.00172	0.00176
N	8	11	8	8	12	11	11	14
Std Error	0.00003	0.00005	0.00004	0.00006	0.00007	0.00005	0.00005	0.00004
**Egg Volume: August** (mm^3)								
Mean	0.00164	0.00160	0.00134	0.00161	0.00174	0.00179	0.00170	0.00173
N	8	9	4	5	13	9	5	8
Std Error	0.00005	0.00006	0.00006	0.00009	0.00006	0.00008	0.00009	0.00007

Table S11: Summary of mixed-model ANOVA testing for the effects of temperature, PAR, UVR, and spawning month on the average egg volume, with genotype as random factor.

	**Egg volume**				
	df	SS	F	p	
Spawn. month	2, 109	2×10^{-7}	7.07	0.001	**
Temperature (T°)	1, 112	7×10^{-7}	46.43	5×10^{-10}	***
UVR	1, 112	3×10^{-8}	1.66	0.201	
PAR	1, 119	7×10^{-8}	4.42	0.038	*
Spawn. month x T°	2, 109	3×10^{-8}	0.87	0.420	
Spawn. month x UVR	2, 109	4×10^{-8}	1.22	0.299	
T° x UVR	1, 112	3×10^{-9}	0.20	0.658	
Spawn. month x PAR	2, 109	4×10^{-8}	1.33	0.270	
T° x PAR	1, 119	1×10^{-8}	0.74	0.390	
UVR x PAR	1, 119	5×10^{-9}	0.29	0.590	
Spawn. month x T° x UVR	2, 109	5×10^{-9}	0.19	0.829	
Spawn. month x T° x PAR	2, 109	4×10^{-8}	1.33	0.269	
Spawn. month x UVR x PAR	2, 109	3×10^{-8}	0.82	0.445	
T° x UVR x PAR	1, 119	2×10^{-9}	0.11	0.736	
Spawn. month x T° x UVR x PAR	2, 109	2×10^{-8}	0.67	0.516	

VI. Other Physiology Characteristics Tables

Table S12: Other physiology characteristics per treatment, showing mean values, number of genotypes (N) and the standard error of the mean

	**High Temperature**				**Low Temperature**			
	**High UVR**		**Low UVR**		**High UVR**		**Low UVR**	
	**High PAR**	**Low PAR**	**High PAR**	**Low PAR**	**High PAR**	**Low PAR**	**High PAR**	**Low PAR**
**Daily Growth**								
Mean[a]	45.93	39.25	49.84	48.82	68.00	64.54	54.25	46.73
	25	25	24	23	23	24	25	25
Std Error	4.01	4.38	4.63	4.87	6.73	6.81	3.81	5.76
**Percent Loss in Pigmentation**								
Mean	17.44	11.10	22.35	19.33	6.62	2.18	12.47	3.39
N	25	25	25	25	25	25	24	25
Std Error	1.91	1.48	1.90	1.79	1.62	1.12	2.13	1.09

Table S13: Summary of mixed-model ANOVA on physiology. Effects on growth. ANOVA conducted on square-root-transformed data to fulfill normality assumptions

	**Growth**				
	df	SS	F	p-value	
Temperature	1, 186	0.033	11.23	0.001	***
UVR	1, 186	0.003	1.00	0.318	
PAR	1, 186	0.007	2.47	0.118	
Temperature x UVR	1, 186	0.026	8.60	0.004	**
Temperature x PAR	1, 186	0.0003	0.09	0.763	
UVR x PAR	1, 186	0.00002	0.01	0.930	
Temperature x UVR x PAR	1, 186	0.003	0.85	0.359	

Table S14: Summary of mixed-model ANOVA on physiology. Effects on discoloration

	**Percent Loss of Pigmentation**				
	df	SS	F	p-value	
Temperature	1, 191	6461	93.92	$< 2 \times 10^{-16}$	***
UVR	1, 191	1267	18.41	3×10^{-5}	***
PAR	1, 191	1617	23.50	3×10^{-6}	***
Temperature x UVR	1, 191	117	1.70	0.194	
Temperature x PAR	1, 191	53	0.77	0.382	
UVR x PAR	1, 191	5	0.07	0.788	
Temperature x UVR x PAR	1, 191	197	2.87	0.092	
